# Towards Improved Collagen Assessment: Polarization-Sensitive Optical Coherence Tomography with Tailored Reference Arm Polarization

**DOI:** 10.1155/2012/892680

**Published:** 2012-03-14

**Authors:** Bin Liu, Christopher Vercollone, Mark E. Brezinski

**Affiliations:** ^1^Department of Orthopedic Surgery, Brigham and Women's Hospital, 75 Francis Street, Boston, MA 02115, USA; ^2^Harvard Medical School, 25 Shattuck Street, Boston, MA 02115, USA

## Abstract

Single channel PS-OCT has advantages for assessing birefringent tissue components in various clinical scenarios, with implications for assessing pathology, ranging from osteoarthritis to myocardial infarction. While the technique has been successfully used both in vitro and in vivo, there have been limited attempts to optimize single channel PS-OCT with respect to performance, particularly paddle rotation. In this study, we developed and tested a new approach for the real-time assessment of birefringence through tailoring of reference arm polarization. Different polarization rotation patterns, as depicted on a Poincare sphere, were assessed with polarization filters and retarders. When further tested in tissue, PS-OCT assessments of bovine cartilage and tendon demonstrated that contrast was sensitive to the pattern selected, indicating that rotation pattern influenced birefringence assessment and providing insights into optimal patterns. We also discuss the difference between diagnostic accuracy and precision with respect to both the construction and application of PS-OCT embodiments.

## 1. Introduction

Assessments of tissue birefringence can be used to identify pathology, as tissue components such as organized collagen are birefringent, and the converse, diseased states, are associated with disorganization [1, 2]. Therefore, techniques for measuring tissue birefringence could improve the evaluation of a range of pathophysiologies. The current paper will investigate a modified approach for the real-time assessment of birefringence through tailored reference arm polarization with the birefringence assessing technology single channel PS-OCT. 

Clinically relevant birefringent tissue components include cholesterol crystals, actin-myosin complexes, nerve fibers (myelin), calcium hydroxyapatite (teeth), and, of particular interest, collagen. Collagen plays a critical role in many pathologic states, either through its depletion or accumulation [3, 4], ranging from osteoarthritis to myocardial infarction. Optical coherence tomography (OCT), an imaging modality based on low coherence interferometry, has demonstrated considerable utility for assessing many tissue pathologies, with up to 1–4 *μ*m resolution while working at or above video rate [5–8]. An OCT subtechnology (adjuvant), polarization-sensitive OCT (PS-OCT), allows assessment of tissue birefringence on a micron scale, in addition to the tissue microstructure [9–11]. With PS-OCT imaging, pathologic microstructural changes in the collagen organization, concentration, and potentially the specific tissue type have been identified [12–15]. This has been demonstrated in humans both *in vitro* and *in vivo*, with results validated against established techniques such as polarization-sensitive microscopy [16–18].

Optical technologies like PS-OCT offer advantages for collagen assessment over other technologies such as MRI due to their relatively high resolution. PS-OCT can be performed with either a single or dual detector/channel setup, the latter having several embodiments. The clinical acceptance of an imaging modality (and most diagnostic technologies) is based on its ability to affect morbidity or mortality, which typically does not correspond to its ability to produce precise numerical data (discrete or continuous). This is the impact of the technology on medicine, not its precision. Instead of numerical data, most clinical analysis with imaging modalities uses categorical information, either nominal or ordinal data, which can be assessed rapidly. When clinical trials are performed, the *categorical data* comparisons are usually compared in terms of specificity and sensitivity, which clinical scientists are most interested in. More relevant, physicians use a Bayesian-like process to assess relative probabilities as data is accumulated, which is best handled categorically and is described in the discussion.

Single-channel PS-OCT looks at relative tissue changes (categorical data), as will be seen for diagnostic accuracy, while dual channel strives for precision and absolute polarization measurement (precise numerical data). But, the vast majority of all clinical imaging is relative assessments/measurements on application (though the technologies have precision design), while precision measurements are uncommon with medical diagnostic technologies. Precision is needed in the design and construction of the technology, but diagnostic accuracy (which usually involves relative measurements) is typically the objective of clinical imaging (in addition to practicality). This is our impression of a gap between medicine and engineering, as physicians are looking for diagnostic accuracy (as well as practicality of use), whereas in engineering, precision is a focus (which is often sacrificed in medicine for other endpoints).

All clinical imaging today strives for clinical accuracy, but this does not mean they strive for precise image information. Look from the perspective of the scrutiny single channel often receives: “Everybody knows absolute numerical values are better than relative values,” this is difficult to defend and a radiograph, ultrasound, or MRI, for example, would never hold to this in practice. The transmission from radiographies of the spin shifts in MRI change dramatically based on patient body size, hydration, temperature, and so forth. But predominately, radiology and MRI assess relative details and not absolute or even numerical data (accurate but not precise), as assessing the heart by either technology is done relative to the lungs and not by absolute numbers. What clinical imaging technology in use operates even close to maximum precision? It is therefore not a surprise that single channel, which is still amenable to improvements such as those in this paper, is arguably more effective than dual channel, even if a dual channel system could ultimately get precise, reliable optical axis information *in vivo*. We strongly favor single detector/channel PS-OCT, the reasons for which we have demonstrated with earlier work [19–22]. From the perspective of our group, the issue of which technology is better, single or dual channel PS-OCT, lies not in which has the highest potential precision, but which is best for addressing a given clinical problem.

The logic stems, in part, from our success in getting real-time answers to specific clinical questions. It also stems from precedent in medicine of relying on the degrees of freedom of an image/data and difference in relative parts of the images. For example, in spite of the vast amount of information on a 12 lead EKG, a cardiologist looking at one typically can identify an acute inferior myocardial infarction in under a few seconds because of pattern recognition, rather than struggling with a list of precision voltages over all segments and leads. Diagnosing pathology in medical imaging, in general, involves using high degrees of freedom and recognizing contrast between regions (similar to single channel PS-OCT). When more quantitative assessments are necessary, this could be potentially achieved with either single or dual channel. Similarly, a radiologist can pick up a lung mass in a chest X-ray in about the same time, in spite of the extensive amount of information contained in the image, in the setting of widely varying body types. While densitometry of the image seems like it could only improve diagnostics by providing more quantitative information, it slows down the process, it is difficult to compensate for artifacts, and it would be without obvious large scale gain.

Our group has been involved in the both the technological advances of OCT as well as its application for over 17 years. Developing OCT technology (as opposed to application), and imaging technology in general, requires precision technology development. If one looks at an example text, “The Introduction to Medical Imaging: Physics, Engineering, and Clinical Application”, the fact that engineering and clinical application sections are approaching problems from a distinct analysis is readily apparent and consistent with our direction for moving forward the technology [23]. In this textbook, the physics and engineering of conventional radiography, CT, MRI, and ultrasound are examined in considerable detail and individually. For all the modalities, from an engineering prospective, the book examines maximizing the technical performance in terms of the point spread function, signal-to-noise ratio, dynamic range, contrast, and so forth (precise numerical data). However, for all the clinical application sections, diagnostic analysis is categorical with extremely few exceptions. And again, we are exploring the basis for superiority claims of dual channel PS-OCT: that its presumed precise numerical data is superior for clinical use compared to the relative or categorical data of the single channel approach.

But if one takes an introductory text like *The Right Imaging Study, a Guide for Physicians*, numerical data is not apparent in any section of the book [24]. And even when numerical data is potentially available, as can be seen, it still is typically presented/examined as ordinal data (absent, mild, moderate, or severe). Common examples include most flow measurements (ultrasound, MRI, or coronary angiography), edema (bone MRI), cartilage breakdown (MRI), and pancreatitis grades (scale 1–5), all of which the available numerical data is presented categorically. It would probably be a surprise to many in the OCT field that even elastography (ultrasound and MRI) and anisotropy for collagen (MRI) are represented by categorical data though numerical data is available. Both OCT elastography and PS-OCT are usually presented in publication s by OCT groups as numerical data, including at times our own. But this is engineering convention, not how it would be used clinically.

Briefly, the single channel approach measures the relative changes in the OCT signal (back-reflection intensity) while the reference arm polarization states are changed, which will be clarified below. The fact that it measures relative changes at points within the tissue improves its robustness over certain artifacts (such as fiber bending) and allows for easy implementation and real-time assessments [22].

Dual channel approaches *attempt* to measure absolute values of tissue birefringence. Although technically more challenging, these techniques offer the potential for measuring certain parameters such as the numerical optical axis, although their clinical utility is unknown at this time. Nonetheless, there may be uncommon clinical scenarios where this precision may be critical. With dual channel approaches, which will be briefly discussed, the Muller matrix is typically measured, or less importantly, the depth-resolved Stokes vectors of the back-reflections from the tissue are measured [25–34]. Most dual channel approaches generating a Muller's matrix of the tissue require analysis of many-frame measurements, and therefore cannot be interpreted rapidly and explicitly. Furthermore, the Stokes or Jones vectors of incident and reflected light, measured at the detector, do not typically represent tissue birefringence properties. This occurs primarily due to birefringence artifacts introduced as the back-reflected light propagates between the sample and detector through variable fiber distortions. Thus, the polarization altering properties of the system need to be maintained constant to prevent artifacts, which adds significant complexity to the system, may not be possible, and is generally not an issue with single channel systems. As we have previously demonstrated, these artifacts can be seen to result in completely opposite results to the actual situation.

This is distinct from the single channel PS-OCT approach, sometimes referred to as single detector PS-OCT, which measures relative birefringence (similar to other clinical imaging technologies which measure relative contrast) and allows clinicians to directly assess birefringence during imaging from the image itself. It is quiescent to catheter or other fiber optic bends in the system because it is measuring birefringence changes relative to different locations in the sample (and not an absolute number at the output) [22]. Our previous work has demonstrated clinically relevant assessments with single channel PS-OCT in tissue including cartilage, tendons, ligaments, and coronary arteries [15–18]. Imaging has been performed *in vivo* in humans, and the validity has been confirmed with picrosirius stained histological sections (polarization microscopy) or scanning electron microscopy (SEM). The data can be obtained in real-time and interpretations are made directly from the image. In an example study of clinical relevance, we have shown that loss of birefringence, which correlates with collagen disorganization, precedes cartilage thinning in osteoarthritis. Similarly, we have confirmed the efficacy of the single channel approach *in vivo* in human knees both in open surgical fields and during arthroscopy [18, 35]. We have also modeled in detail the theoretical principles behind the ability of single channel PS-OCT to measure tissue birefringence [20].

However, as will be seen in this work, the single channel PS-OCT technique to date has never been optimized with respect to the reference arm polarization rotation, which could further improve the performance. For birefringent tissue, the back-reflection intensity changes with alterations of the reference arm polarization state. The greater the birefringence, the more rapidly the back-reflection changes with the reference arm polarization alterations. The birefringence can be characterized by interframe back-reflection changing in the reference arm, but no study has been systematically conducted on how best to rotate polarization in the reference arm.

In this work, we hypothesize that single channel PS-OCT can be optimized by tailoring of the reference arm polarization rotation. The relative position and rate of paddle alterations in the reference arm dictate the pattern of sample back-reflection change. Different patterns (with different paddle rotations) may yield superior birefringence measurements and thus we will first plot different patterns along Poincare spheres for analysis. The influence of different paddle modulation (and movement through the Poincare sphere) on collagen measurement in tissue will then be followed to illustrate the principles.

## 2. Methods

**Figure 1:**
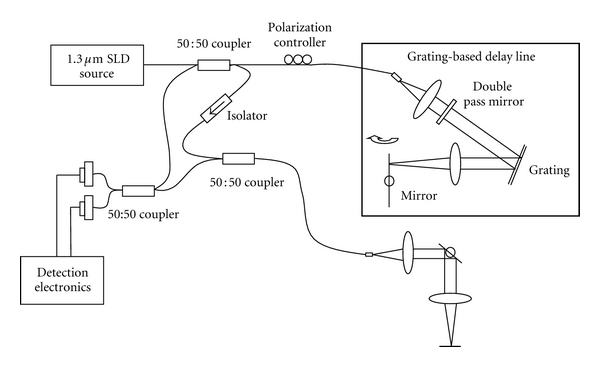
Schematics of the single channel PS-OCT for the experimental investigation.

The general optical arrangement for a single channel PS-OCT is depicted in Figure 1. The system used here is time domain OCT (TD-OCT), but swept source (SS-OCT) could be used without loss of generality. The system uses a Michelson-type fiber optic interferometer built with a 2 × 2 fiber coupler. A broadband superluminescent diode (SLD) source with 1300 nm center wavelength, 60 nm full-width-half-maximum (FWHM), and 12 mW output power (AFC, Toronto, Canada) was used. A grating-based delay line was used in the reference arm, providing longitudinal scans (A-scans). The light beam in the sample arm was focused on the tissue sample and scanned laterally by a galvanometer-driven scanner, providing lateral scans (B-scans). The polarization controller in the reference arm was a standard Lefevre-type fiber optic polarizer [34]. It consists of a certain length of single mode fiber spooled on three paddles. Two-quarter wave coils control the ellipticity and a half wave coil controls linear states. 

As shown in Figure 1, the interferograms from the two ports of the coupler are guided into a dual-balanced detector (New Focus, 2117, Santa Clara, CA) from the circulator and another coupler, respectively; this is standard to remove photon excess noise. Our previous work demonstrates that, in a single channel PS-OCT, the irradiance at the detector, for a given paddle position, is described by [20]:

(1) $$\begin{array}{cc} I_{D}\left(z_{R}\right) & =I_{R}+I_{S}\\ & \;+\frac{I_{0}}{4}\{2\cos\left[2\pi k_{0}\Delta n_{S}\left(\omega_{0}\right)z_{R}\right]M_{y}\left(\frac{z_{R}}{n_{S}\left(k_{0}\right)}\right)\\ & \;\;\;+\left[M_{x}\left(\frac{z_{R}}{n_{S}\left(k_{0}\right)}\right)-M_{y}\left(\frac{z_{R}}{n_{S}\left(k_{0}\right)}\right)\right]\}\\ & \;⊗\left\{\cos\left[4\pi k_{0}z_{R}\right]\exp\left[-\frac{4\ln2z_{R}^{2}}{\Delta_{l}^{2}}\right]\right\}, \end{array}$$

where *k*_0_ is known as the central wavenumber of the light source, Δ*n*_*S*_(*k*_0_) is the difference of the principle indices, and Δ* l* represents the full-width half maximum (FWHM) coherence length of the light source. Bedsides the longitudinal back-reflection profile *p*_*S*_(*z*), the interferogram includes the depth-resolved tissue birefringence information. The depth-integrated retardation determined by Δ*n*_*S*_ is encoded in the sinusoidal-type modulation, which corresponds to the banding pattern in the single-detector PS-OCT image from a birefringent tissue. Optic axis variations of the tissue, combined with the polarization state in the reference arm (the paddle position), are encoded in the *z*-dependent modulation functions *M*_*x*_(*z*) and *M*_*y*_(*z*). Continuously rotating the paddles modulates the interferograms and the tissue birefringence can be assessed by the changes. 

The maximum interferogram intensity requires polarization to be the same in the reference and sample arm. We will use that fact to establish polarization state in the reference arm by interfering it with light in the sample arm, which contains a mirror and polarization analyzers. The procedure is described here. The first step of the experiment was determining the polarization states in the reference arm while the paddles were rotated. The Stokes vector of the returned reference beam was measured from the interferograms, at each paddle position, with polarization analyzers placed in the sample arm above a mirror. Results in different paddle positions were plotted onto the Poincare sphere and displayed as a trajectory. Specifically, at each paddle position, a linear polarizer was placed above a mirror in the sample arm and rotated at 0, 90, 45, and −45 degree mechanical angles, consecutively. Meanwhile, a neutral density (ND) attenuator was adjusted to normalize the return power from the sample arm (reduce back-reflection variability from different polarizers). The corresponding interferogram was then recorded and the signal intensity (peak-to-peak value) calculated. Next, while maintaining the paddle position, a quarter wave plate was inserted into the sample arm. Its fast axis was aligned at 45 and −45 degree with respect to the linear polarizer for obtaining the right and left circular polarization state in the sample arm. The interferogram was measured as described above. Each set of measurements (interferograms at a given paddle position) was composed of six components: *V*_0_, *V*_90_, *V*_45_, *V*_−45_, *V*_RC_, and *V*_LC_, which were in units of volts. Again, to avoid confusion, we are doing this to optimize the reference arm for use; this is not intended to be done each time clinical imaging is performed. For calculating the Stokes vector, the following conversions were followed: *I*_0_ = V_0_^2^, *I*_90_ = *V*_90_^2^, *I*_45_ = *V*_45_^2^, *I*_−45_ = *V*_−45_^2^, *I*_RC_ = *V*_RC_^2^, and *I*_LC_ = *V*_LC_^2^. Then, the Stokes vector was calculated by: S0 = [(*I*_0_ + *I*_90_)+(*I*_45_ + *I*_−45_)+(*I*_RC_ + *I*_−LC_)]/3; *S*1 = (*I*_0_  –  *I*_90_)/(*I*_0_ + *I*_90_); *S*2 = (*I*_45_–*I*_−45_)/( *I*_45_ + *I*_−45_); *S*2 = (*I*_*RC*_ − *I*_LC_)/(*I*_RC_ + *I*_−LC_). At each reference arm paddle position, the Stokes vectors were measured and displayed on the Poincare sphere.

With our use of the Poincare sphere, we are trying to obtain two objectives: (1) determine if different patterns on the sphere result in improved contrast; (2) a way of standardizing the reference arm so we can repeatedly put it into the same or *near* the same optimal state. We have achieved both of these. With regard to this, two questions arise:  (1) if fiber bending or temperature changes are occurring in the reference arm, is not it true that the point on the sphere is not the same as that directly coming off the mirror or returning to the beam splitter; (2) what happens with fiber bending in the sample arm. These are addressed in the discussion.

Once the polarization states of paddle positions in the reference arm were determined through different rotation profiles, PS-OCT imaging was performed of bovine tendon and cartilage using different paddle rotations. In other words, how imaging of samples was affected by altering polarization along different paths on the sphere was evaluated. Tissue birefringence was assessed as changing back-reflection intensity over a region of interest (ROI). A ROI was selected as a rectangular area in each OCT image frame (100 × 20 pixel, H × W), at a location near the middle portion, about 400microns under the surface. While the paddle was rotating, the average signal intensity over the ROI was examined and compared with the same parameter over the same area within the serial images obtained at different paddle positions. 

## 3. Results

**Figure 2:**
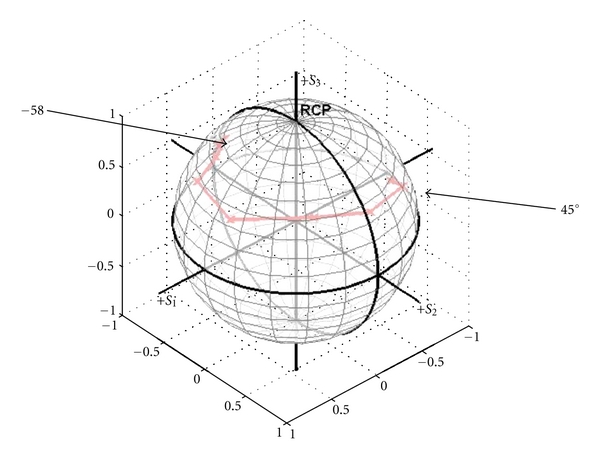
The trajectory of Stokes vectors on the Poincare sphere, while the paddle in the reference arm was manually rotated from angle of −58° to 45° with roughly 15 degrees increment.

Figures 2 through 4 reflect assessments done with a mirror and polarization analyzers in the sample arm. Figures 5 and 6 show assessments with tissue.  Figure 2 demonstrates a plot of reference arm states on the Poincare sphere when the paddles are moved from −58° to 45° mechanical degrees with roughly 15 degree increments. It can be seen that as the paddle position in the reference arm is changing, the Stokes vector of the reference beam changes and walks on the sphere, which demonstrates that the polarization states of the reference arm are tractable. 

**Figure 3:**
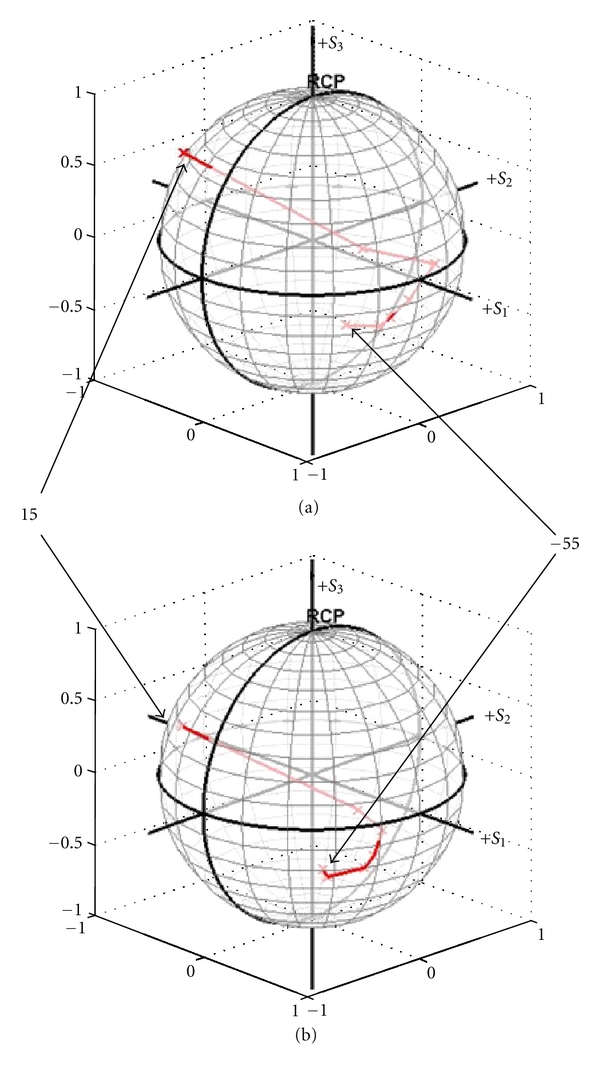
Trajectories of Stokes vectors of the reference beam from two repeated paddle maneuver processes.

Reproducibility of measurements is examined in Figure 3. A. The paddle was rotated in a fixed pattern as measurements are made of the polarization state and then plotted on the Poincare sphere. The same paddle rotation pattern was then repeated after 8 days. Best fix analysis shows no significant difference between the two curves, supporting reproducibility of the technique. 

**Figure 4:**
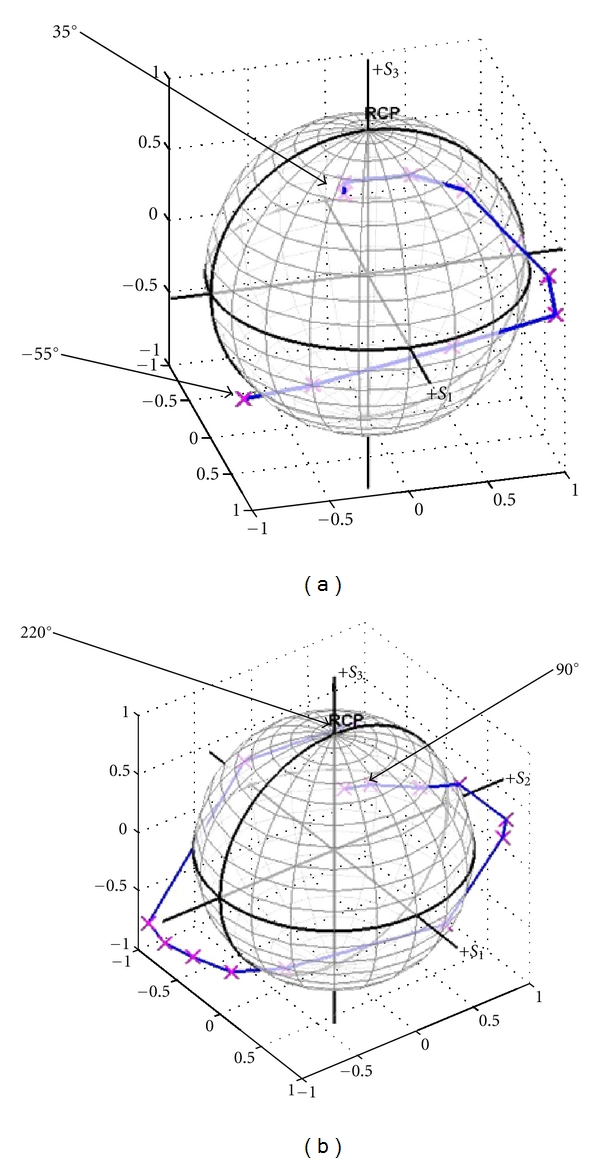
Stokes vectors of the reference beam (magenta markers) and their trajectories (blue lines) on the Poincare sphere, at different paddle positions: (a) from angle of −55° to 35°, with 10° increment; (b) from 90° to 220° with the same angular increment.

**Figure 5:**
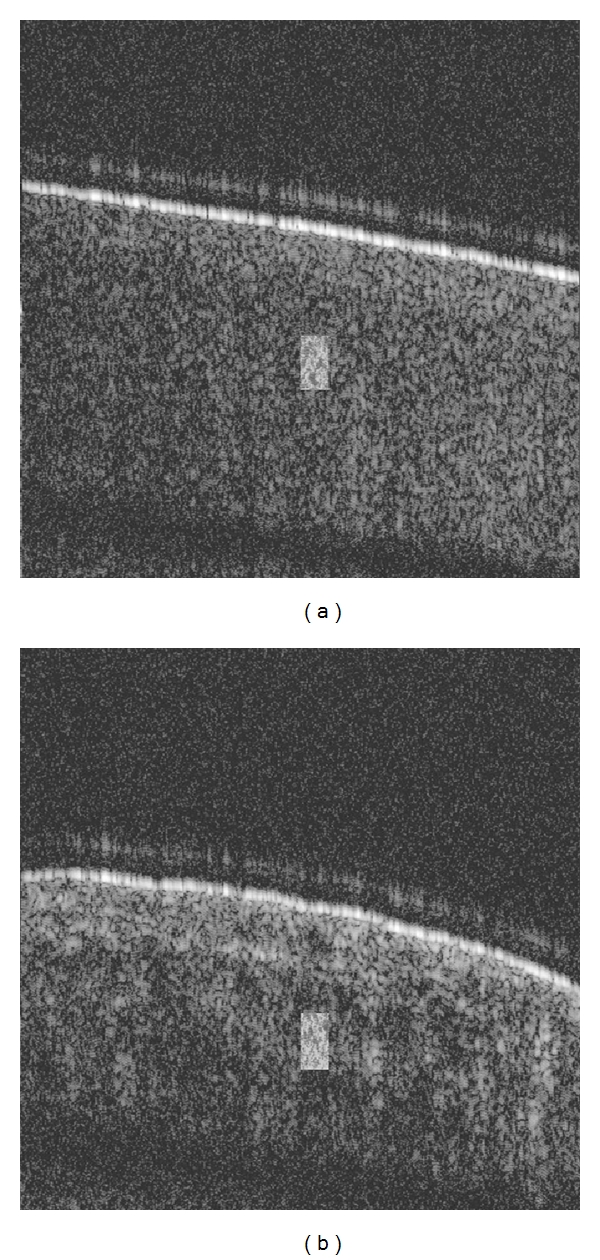
Sample images of a bovine cartilage (a), and a bovine tendon (b), when the angle of the paddle was set at −15° from the plane of the optical table.

**Figure 6:**
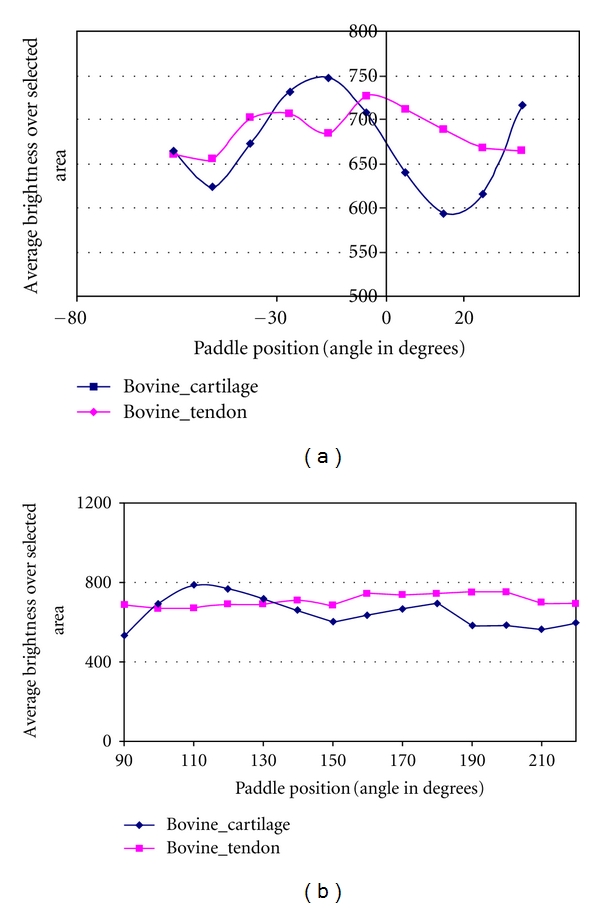
The results of the average signal intensity of ROI vs. paddle position in graphic: (a) from −55° to 35° in mechanical angle with 10° increment; (b) from 90° to 220° with the same angular increment.

Figures 5 and 6 examine two different reference arm polarization rotation patterns for optimization of contrast within biological tissues. In Figure 4 these rotation patterns are demonstrated on the Poincare sphere. Specifically, Figure 4 compares polarization state changes when the paddle rotates through two different patterns: (a) from −55° to 35° paddle degree, and (b) from 90° to 220°, with 10° increments for each. Curve (pattern) (a) stays closer to the linear polarization state plane than curve (b). The intensities of the sample beam at any given polarizer position were maintained within the range of ±2% around the set level. This is in spite of the condition that the polarizer and quarter wave plate measurement angle was produced manually, which typically is considered the major source of the Stokes vector measurement errors. 

Figure 5 represents images of bovine cartilage (a), and bovine tendon (b) by single-detector PS-OCT imaging where in this case the angle of the paddle was set at −15°. 

Figure 6 plots the average signal intensity over the region of interest (ROI) in bovine cartilage and tendon images against the paddle position in the reference arm, rotated from: (a) −55° to 35° and (b) from 90° to 220°. The 100 × 20 pixel (H × W) ROI was selected in each image as inversely highlighted as shown in Figure 5. The average signal intensity was examined over this area. It can be seen that the rotation pattern introduced as Figure 4(a) (relatively linear polarization states) more sharply differentiates between the tissue types (contrast) as shown in (a) rather than the pattern shown in Figure 4(b). Again, the major difference between the pattern of Figures 4(a) and 4(b) is that the former has movement in the S_1_-S_2_ direction and less in the S_3_ (circular polarization axis), demonstrating that differences do exist which allow optimization of imaging. 

## 4. Discussion

Birefringent biomolecules include collagen, enamel, dentin, nervous tissue, and actin-myosin complexes. Many pathophysiologies regarding these molecules are important in diseases such as myocardial infarction, osteoarthritis, and dental caries. Polarization-sensitive OCT (PS-OCT) has demonstrated feasibility in assessing the presence of these birefringent biomolecules and pathological states. This paper seeks and does improve the capabilities of single channel PS-OCT by using a tailored reference arm.

PS-OCT can be categorized as either single or dual channel, with various subcategories and definite distinctions between the two. The issue of which technology is better, single or dual channel PS-OCT, lies not in which is superior, but rather, which is best for the appropriate problem. In constructing the technology, precision is critical, but for clinical use, diagnostic accuracy and practicality are essential.

As stated, physicians in general use a Bayesian-like process for assessing patients where a single test is rarely diagnostic. A patient comes in with a set of symptoms, physical exam results, and initial diagnostic tests. Initial diagnostic probabilities are developed and are modified by incoming data. Now, let us say there is categorical temperature spike (it could be a 101.5°F or 103°F, precision does not matter), it alters the diagnostic probabilities. The same would result if blood glucose were 200, 300, or 400. These all fall into the same categorical data and would affect the diagnostic probability. If we take the case of temperature, physicians still generally prefer mercury thermometers (removed because of mercury toxicity and glass) over the “more sophisticated” digital data in critical situations because even though the latter gives higher precision data, the simpler former method is associated with less application artifact.

For over a decade, we have tailored our PS-OCT to address clinical need, not the question for more precise numerical data. For analytical chemistry or measuring low level background radiation in space, precision is essential. Engineering and medical approaches are the right ones for their respective fields, as long histories have demonstrated. For medicine, categorical data with high sensitivity and specificity to be integrated into a Bayesian-like thought process is the how the field operates. This, along with technical advantages, is why we strongly prefer single channel PS-OCT over dual channel. The logic stems, in part, from our success in testing hypotheses under *in vitro* and *in vivo* clinical scenarios using this technology. It also stems in part from precedent in medicine, where the advantages of single channel PS-OCT make it more similar in function to other clinical imaging modalities as compared to dual channel.

Single channel PS-OCT is a technology which for a considerable period of time has demonstrated the ability to identify pathology *in vivo* and *in vitro*. In this paper, we study further improving the contrast of the technology. Yet much of the field believes that not just precision in the technology is needed, but precision in measurement parallels clinical accuracy. Almost all clinical imaging is based on relative differences in images and not obtaining precise numerical values. For those who have not practiced medicine, it is understandable why this point would not be obvious. But, being allowed to deviate to a nonmedical example, suppose that 1000 feet away in an open field was a lion. The question of major concern is the lion moving toward me, stationary, or moving away. It would also be useful to know if he was moving, whether he was walking or running. The observer is using Bayesian-like logic, so he is considering other parameters such as proximity to a car, terrain, and so forth. But, we will ignore these other parameters without loss of generality to simplify the analogy. The observer has a pair of binoculars and an optically based velocity meter. Now we want both to be built to maximum precision. But to answer the question needed, pulling the binoculars out of bag, getting a quick answer (general running versus walking can be readily and easily assessed by relative movement of limbs and with respect to the ground) is the optimum strategy to decide to run. However, an observer may decide on the velocity meter, wanting to know with high precision how fast the lion is moving (to three significant digits). The use of this technology involves considerably more effort and time. In addition, it can be dramatically influenced by many sources of error including environmental factors or even accidentally measuring on the velocity of something near the lion rather than the lion itself (getting a velocity of zero). Errors of this type in making rapid decisions are highly unlikely with the binoculars. Hopefully this crude example serves as an analogy to the clinical imaging process.

The specific additional advantages of single channel PS-OCT (relative imaging modality) are numerous. First, the system is less expensive, which is important for routine clinical use. Second, the changes in the image of the single channel system are easy to interpret on screen as a change in backscattering intensity as the polarization of the incident light is changed. Again, this is part of being a relative imaging technology analogous to radiography, MRI, CT, and ultrasound. Third, in the dual detector system, multiple polarization filters and beam splitters are integrated into the system, leading to a reduction of power incident on the sample. Since portable sources such as quantum well devices or doped fibers have limited power, the addition of these filters into the OCT system may result in power levels dropping below what is necessary for *in vivo* use. Finally, dual detector approaches suffer from noise issues when most of the power falls on one detector. Single channel approaches generally measure peak-to-peak intensities so detector noise is a trivial issue, which is in sharp contrast to the asymmetric power problem of dual channel.

With a superiority in managing certain birefringence artifacts, the single channel design allows an ease of use and implementation that results in clinically advantageous real-time measurements, as opposed to more the complex dual channel modality. Single channel PS-OCT has already been successfully applied to imaging human tissue *in vitro and in vivo*, most notably cartilage and coronary arteries, in addition to animal models of disease.

 In our approach, single channel PS-OCT utilizes the changing of reference arm polarization states in a traditional OCT system to perform diagnostics. It looks at how back-reflection intensity changes as a function of polarization paddle changes. However, this approach has not yet been optimized with respect to the states through which the reference arm is rotated. We developed and tested a new approach for the real-time assessment of birefringence through tailored reference arm polarization. Results from imaging bovine cartilage and tendon have confirmed that by controlling the reference arm polarization-changing pattern, we can improve the assessment of tissue collagen organization, particularly in tissues with relatively low birefringence. Increased signal intensity and increased ability for differentiation between tissue types was found using a −35° to 55° range, which corresponds to increased movement in the x-y direction rather than the z direction. This not only proves that the optimization of reference arm polarization states does in fact provide increased ability for sample analysis, but gives some base for how that optimization might occur.

In this approach, paddle rotation was achieved manually and the birefringence contrast was evaluated offline and demonstrated graphically. By using motorized paddle controllers, fiber squeezers, or similar techniques in conjunction with software detection schemes, future systems will be even more clinically friendly.

With respect to the first question, this may be possible but the likelihood it is relevant is small. This is not dual channel PS-OCT and absolute values are not needed. The sphere plot is representative of a pattern at the beam splitter and does not need to be precisely the pattern of the end target. In other words, the actual end target plots can be slightly different, but a dual channel approach would be essentialy unusable under the same conditions. Again, this is an advantage of a technique which relies on relative rather than attempted absolute imaging. If fiber bending does cause some degree of shift, it is a shift of the entire pattern, which is the phase change. This shifts the peak but does not alter peak to peak distances or contrast, so it is not significant. Objectives one and two, which are the focus of the paper, are still maintained. We are optimizing contrast for assessing pathology, not trying to get precise target information. To the second point (will fiber bending during a procedure affect results), it is unlikely to be a significant issue similarly to our previous paper on fiber bending. From a practical standpoint fiber bends tend to occur very slowly. A catheter introduced in the coronary artery, for example, tends not to bend once in the artery. But even when bends are occurring in other clinical scenarios, they tend to occur at time intervals much longer than the scanning interval. In addition, we are measuring relative birefringence. Therefore, as above, the birefringence would tend to rotate or shift the pattern on the Poincare sphere, but its shape would remain relatively unchanged. This is analogous to identifying a lung mass by difference of contrast between the lung and the mass. In diagnostics which use relative measurements (which represents the majority of imaging), assessments are only minimally affected by, for example, changes in body size, whereas techniques attempting measurement of an absolute value like dual channel are highly vulnerable to these artifacts [22].

## 5. Conclusion

This paper demonstrates a new approach to single channel PS-OCT which tailors the reference arm changing polarization pattern. The paper also addresses the concept that in optics and engineering of systems high precision is usually critical. However, when applying the technology for clinical diagnostics, clinical accuracy and contrast are likely far more important as physicians use categorical data and Bayesian-like logic in the decision processes. This has been the case in our clinical application of the technology.
